# 4,4-Diacetyl­hepta­nedinitrile

**DOI:** 10.1107/S1600536808039962

**Published:** 2008-12-03

**Authors:** Guo-wei Wang, Jian Zhang, Ling-hua Zhuang, Wen-yuan Wu, Jin-tang Wang

**Affiliations:** a Department of Light Chemical Engineering, College of Science, Nanjing University of Technology, Nanjing 210009, People’s Republic of China; b Department of Applied Chemistry, College of Science, Nanjing University of Technology, Nanjing 210009, People’s Republic of China

## Abstract

The asymmetric unit of the title compound, C_11_H_14_N_2_O_2_, contains one half-mol­ecule as the central C atom of the mol­ecule lies on a twofold rotation axis. In the crystal structure, weak inter­molecular C—H⋯N hydrogen bonds link the mol­ecules into zigzag chains along *c*.

## Related literature

For details of the biological activity of amino­thia­zoles, see: Kabalka & Mereddy (2006 ▶). For their use in organic synthesis, see: Kim *et al.* (2001 ▶); Ranu & Banerjee (2005 ▶); Ranu *et al.* (2006 ▶); Wang *et al.* (2008 ▶). For bond-length data, see: Allen *et al.* (1987 ▶).
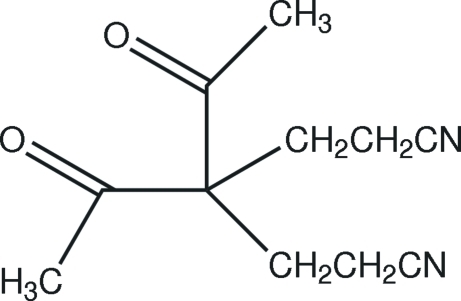


## Experimental

### 

#### Crystal data

C_11_H_14_N_2_O_2_
*M* *_r_* = 206.24
Monoclinic, 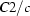

*a* = 12.562 (3) Å
*b* = 7.8700 (16) Å
*c* = 10.941 (2) Å
β = 84.91 (3)°
*V* = 1077.4 (4) Å^3^
*Z* = 4
Mo *K*α radiation
μ = 0.09 mm^−1^
*T* = 293 (2) K
0.30 × 0.20 × 0.10 mm

#### Data collection

Enraf–Nonius CAD-4 diffractometer
Absorption correction: ψ scan (North *et al.*, 1968 ▶) *T*_min_ = 0.961, *T*_max_ = 0.991
1009 measured reflections
974 independent reflections
758 reflections with *I* > 2σ(*I*)
*R*_int_ = 0.024
3 standard reflections every 200 reflections intensity decay: 9%

#### Refinement

*R*[*F* ^2^ > 2σ(*F* ^2^)] = 0.071
*wR*(*F* ^2^) = 0.152
*S* = 1.00
974 reflections
70 parameters
H-atom parameters constrained
Δρ_max_ = 0.27 e Å^−3^
Δρ_min_ = −0.20 e Å^−3^

### 

Data collection: *CAD-4 Software* (Enraf–Nonius, 1989 ▶); cell refinement: *CAD-4 Software*; data reduction: *XCAD4* (Harms & Wocadlo, 1995 ▶); program(s) used to solve structure: *SHELXS97* (Sheldrick, 2008 ▶); program(s) used to refine structure: *SHELXL97* (Sheldrick, 2008 ▶); molecular graphics: *SHELXTL* (Sheldrick, 2008 ▶); software used to prepare material for publication: *SHELXTL*.

## Supplementary Material

Crystal structure: contains datablocks global, I. DOI: 10.1107/S1600536808039962/sj2557sup1.cif

Structure factors: contains datablocks I. DOI: 10.1107/S1600536808039962/sj2557Isup2.hkl

Additional supplementary materials:  crystallographic information; 3D view; checkCIF report

## Figures and Tables

**Table 1:** Hydrogen-bond geometry (Å, °)

*D*—H⋯*A*	*D*—H	H⋯*A*	*D*⋯*A*	*D*—H⋯*A*
C6—H6*B*⋯N^i^	0.97	2.66	3.533 (5)	150

Symmetry code: (i) 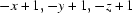.

**Fig. 1.**
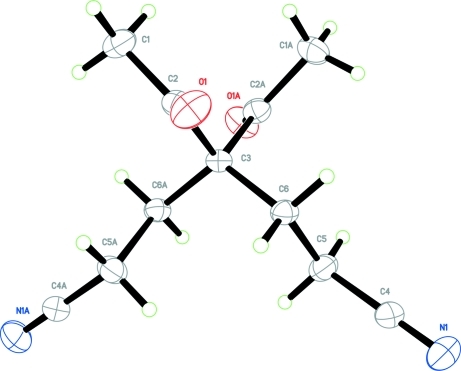
A view of the molecular structure of (I) showing the atom-numbering scheme and 30% displacement ellipsoids (arbitrary spheres for the H atoms).

**Fig. 2.**
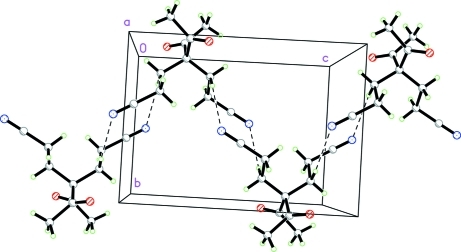
The crystal packing of (I), viewed down the a axis. Hydrogen bonds are drawn as dashed lines.

## supplementary crystallographic information

### Comment

The biological activity of aminothiazoles has been well documented. They have broad applications in the treatment of allergies, hypertension, schizophrenia,inflammation, bacterial infections, and HIV (Kabalka & Mereddy, 2006). Dicarbonyl compounds represent an important class of starting materials materials used to increase the carbon number of organic compounds (Kim *et al.*, 2001). Many dicarbonyl compounds have been synthesized by the Michael addition method using diethyl malonate as starting compound, but only a few Michael addition diadducts were synthesized under normal conditions (Ranu & Banerjee, 2005; Ranu *et al.*, 2006). We are focusing our synthetic and structural studies on new products of Michael addition reactions from dicarbonyl compounds (Wang *et al.*,2008) and we report here the crystal structure of the title compound (I), Fig. 1.

All bond lengths are within normal ranges (Allen *et al.*, 1987). The asymmetric unit contains one half-molecule, and the central C4 atom lies on a twofold rotation axis at right angles to the *ac* plane, which generates the other half-molecule.  In the crystal structure weak, intermolecular C6—H6B···N  hydrogen bonds link the molecules into zig-zag chains along the *c* axis, Table 1, Fig 2.

### Experimental

2,4-Pentanedione (50 mmol) was dissolved in n-hexane (40 ml) and anhydrous potassium carbonate (100 mmol) and tetrabutylammonium bromide (0.5 g) added. Acrylonitrile (100 mmol) was added dropwise to this solution and the mixture refluxed for 6 h. 50 ml ethyl acetate were then added, the organic layer was filtered and the solvent removed under vacuum to yield the crude product (I). This was crystallized from ethyl acetate (15 ml). Crystals of (I) suitable for X-ray diffraction were obtained by slow evaporation of acetonitrile.

### Refinement

All H atoms were positioned geometrically, with C—H = 0.96 and 0.97 Å for methyl and methylene H atoms, and constrained to ride on their parent atoms, with *U*_iso_(H) = *xU*_eq_(C), where *x*= 1.5 for methyl H and *x* = 1.2 for methylene H atoms.

### Crystal data

**Table:**

C_11_H_14_N_2_O_2_	*F*(000) = 440
*M**_r_* = 206.24	*D*_x_ = 1.271 Mg m^−^^3^
Monoclinic, *C*2/*c*	Mo *K*α radiation, λ = 0.71073 Å
Hall symbol: -C 2yc	Cell parameters from 25 reflections
*a* = 12.562 (3) Å	θ = 10–13°
*b* = 7.8700 (16) Å	µ = 0.09 mm^−^^1^
*c* = 10.941 (2) Å	*T* = 293 K
β = 84.91 (3)°	Block, colourless
*V* = 1077.4 (4) Å^3^	0.30 × 0.20 × 0.10 mm
*Z* = 4	

### Data collection

**Table:**

Enraf–Nonius CAD-4 diffractometer	758 reflections with *I* > 2σ(*I*)
Radiation source: fine-focus sealed tube	*R*_int_ = 0.024
graphite	θ_max_ = 25.3°, θ_min_ = 3.1°
ω/2θ scans	*h* = −14→15
Absorption correction: ψ scan (North *et al.*, 1968)	*k* = 0→9
*T*_min_ = 0.961, *T*_max_ = 0.991	*l* = 0→13
1009 measured reflections	3 standard reflections every 200 reflections
974 independent reflections	intensity decay: 9%

### Refinement

**Table:**

Refinement on *F*^2^	Primary atom site location: structure-invariant direct methods
Least-squares matrix: full	Secondary atom site location: difference Fourier map
*R*[*F*^2^ > 2σ(*F*^2^)] = 0.071	Hydrogen site location: inferred from neighbouring sites
*wR*(*F*^2^) = 0.152	H-atom parameters constrained
*S* = 1.00	*w* = 1/[σ^2^(*F*_o_^2^) + (0.0339*P*)^2^ + 4.1549*P*] where *P* = (*F*_o_^2^ + 2*F*_c_^2^)/3
974 reflections	(Δ/σ)_max_ < 0.001
70 parameters	Δρ_max_ = 0.27 e Å^−^^3^
0 restraints	Δρ_min_ = −0.19 e Å^−^^3^

### Special details

**Table:**

Experimental. ^1^H NMR (DMSO, δ, p.p.m.) 2.15 (s, 6H), 2.23 (t, 4H), 2.31(t, 4H).
Geometry. All e.s.d.'s (except the e.s.d. in the dihedral angle between two l.s. planes) are estimated using the full covariance matrix. The cell e.s.d.'s are taken into account individually in the estimation of e.s.d.'s in distances, angles and torsion angles; correlations between e.s.d.'s in cell parameters are only used when they are defined by crystal symmetry. An approximate (isotropic) treatment of cell e.s.d.'s is used for estimating e.s.d.'s involving l.s. planes.
Refinement. Refinement of *F*^2^ against ALL reflections. The weighted *R*-factor *wR* and goodness of fit *S* are based on *F*^2^, conventional *R*-factors *R* are based on *F*, with *F* set to zero for negative *F*^2^. The threshold expression of *F*^2^ > σ(*F*^2^) is used only for calculating *R*-factors(gt) *etc*. and is not relevant to the choice of reflections for refinement. *R*-factors based on *F*^2^ are statistically about twice as large as those based on *F*, and *R*- factors based on ALL data will be even larger.

### Fractional atomic coordinates and isotropic or equivalent isotropic displacement parameters (Å^2^)

**Table:**

	*x*	*y*	*z*	*U*_iso_*/*U*_eq_	
O	0.65270 (17)	−0.0258 (3)	0.3475 (2)	0.0523 (7)	
N	0.3630 (3)	0.4652 (4)	0.5714 (3)	0.0646 (9)	
C1	0.6246 (3)	−0.1760 (4)	0.1653 (3)	0.0495 (9)	
H1A	0.6530	−0.1210	0.0912	0.074*	
H1B	0.5608	−0.2368	0.1503	0.074*	
H1C	0.6765	−0.2541	0.1920	0.074*	
C2	0.5988 (2)	−0.0454 (4)	0.2629 (3)	0.0369 (7)	
C3	0.5000	0.0686 (5)	0.2500	0.0288 (8)	
C4	0.3719 (2)	0.3939 (4)	0.4802 (3)	0.0442 (8)	
C5	0.3865 (3)	0.3008 (4)	0.3639 (3)	0.0440 (8)	
H5A	0.4002	0.3802	0.2966	0.053*	
H5B	0.3218	0.2383	0.3511	0.053*	
C6	0.4802 (2)	0.1776 (4)	0.3665 (2)	0.0341 (7)	
H6A	0.4670	0.1029	0.4367	0.041*	
H6B	0.5445	0.2421	0.3777	0.041*	

### Atomic displacement parameters (Å^2^)

**Table:**

	*U*^11^	*U*^22^	*U*^33^	*U*^12^	*U*^13^	*U*^23^
O	0.0447 (12)	0.0599 (15)	0.0551 (13)	0.0160 (11)	−0.0197 (10)	−0.0091 (12)
N	0.072 (2)	0.0551 (19)	0.0633 (19)	0.0016 (17)	0.0109 (15)	−0.0178 (17)
C1	0.0502 (19)	0.0408 (19)	0.057 (2)	0.0116 (15)	−0.0038 (15)	−0.0075 (16)
C2	0.0355 (15)	0.0341 (16)	0.0418 (16)	0.0002 (13)	−0.0073 (12)	0.0048 (13)
C3	0.0305 (18)	0.0244 (19)	0.0319 (19)	0.000	−0.0057 (15)	0.000
C4	0.0466 (17)	0.0325 (16)	0.0519 (19)	0.0021 (14)	0.0048 (14)	−0.0001 (15)
C5	0.0497 (18)	0.0375 (17)	0.0440 (17)	0.0072 (14)	−0.0005 (13)	−0.0057 (14)
C6	0.0423 (15)	0.0281 (15)	0.0324 (14)	0.0013 (12)	−0.0058 (11)	0.0006 (12)

### Geometric parameters (Å, °)

**Table:**

O—C2	1.205 (3)	C3—C6	1.538 (3)
N—C4	1.142 (4)	C4—C5	1.466 (4)
C1—C2	1.497 (4)	C5—C6	1.528 (4)
C1—H1A	0.9600	C5—H5A	0.9700
C1—H1B	0.9600	C5—H5B	0.9700
C1—H1C	0.9600	C6—H6A	0.9700
C2—C3	1.547 (3)	C6—H6B	0.9700
			
C2—C1—H1A	109.5	N—C4—C5	178.3 (4)
C2—C1—H1B	109.5	C4—C5—C6	109.8 (3)
H1A—C1—H1B	109.5	C4—C5—H5A	109.7
C2—C1—H1C	109.5	C6—C5—H5A	109.7
H1A—C1—H1C	109.5	C4—C5—H5B	109.7
H1B—C1—H1C	109.5	C6—C5—H5B	109.7
O—C2—C1	122.3 (3)	H5A—C5—H5B	108.2
O—C2—C3	120.4 (3)	C5—C6—C3	114.0 (2)
C1—C2—C3	117.2 (2)	C5—C6—H6A	108.8
C6—C3—C6^i^	112.2 (3)	C3—C6—H6A	108.8
C6—C3—C2^i^	109.09 (15)	C5—C6—H6B	108.8
C6—C3—C2	108.63 (15)	C3—C6—H6B	108.8
C2^i^—C3—C2	109.2 (3)	H6A—C6—H6B	107.6
			
O—C2—C3—C6	−7.9 (4)	C1—C2—C3—C2^i^	54.4 (2)
C1—C2—C3—C6	173.3 (3)	C4—C5—C6—C3	178.0 (2)
O—C2—C3—C6^i^	114.6 (3)	C6^i^—C3—C6—C5	57.5 (2)
C1—C2—C3—C6^i^	−64.2 (3)	C2^i^—C3—C6—C5	−62.9 (3)
O—C2—C3—C2^i^	−126.8 (3)	C2—C3—C6—C5	178.1 (2)

Symmetry codes: (i) −*x*+1, *y*, −*z*+1/2.

### Hydrogen-bond geometry (Å, °)

**Table:**

*D*—H···*A*	*D*—H	H···*A*	*D*···*A*	*D*—H···*A*
C6—H6B···N^ii^	0.97	2.66	3.533 (5)	150

Symmetry codes: (ii) −*x*+1, −*y*+1, −*z*+1.
